# Analyzing referencing patterns in grey literature produced by influential global management consulting firms and international organizations

**DOI:** 10.1371/journal.pone.0279723

**Published:** 2023-02-28

**Authors:** Sumayya Saleem, Elizabeth Dhuey, Linda White, Jamie Waese, Michal Perlman

**Affiliations:** 1 Ontario Institute for Studies in Education, Applied Psychology and Human Development, University of Toronto, Toronto, Ontario, Canada; 2 Department of Management, University of Toronto, Toronto, Ontario, Canada; 3 Department of Political Science, University of Toronto, Toronto, Ontario, Canada; 4 BAM! Global Industries, Toronto, Ontario, Canada; University of Siena, Italy, ITALY

## Abstract

Given the growing influence of non-academic organizations in the policy sphere, it is important to investigate the evidence both produced by and relied on by these organizations. Using citation analysis, a methodology primarily used in academic literature, we investigated the evidence base supporting the grey literature published by leading global management consulting firms (GMCFs) and international organizations (IOs). With the topic of the skills needed for the future of work as a case study, we collected 234 reports published by influential GMCFs and IOs over twenty years. By extracting references from the bibliographies of these reports we: 1) analyzed referencing patterns by measuring citation counts, institutional self-referencing and utilization of scholarly sources; 2) compared reference patterns across GMCFs and IOs; and 3) described the most influential sources. Overall, both GMCFs and IOs showed increasing reliance on grey literature, demonstrated high levels of self-referencing, and had considerable variation in the number of sources referred to. Across type of publishing organization, we found that IOs had better referencing practices than GMCFs. Our findings call into question the evidence-base behind the reports published by these policy actors. We emphasize the need to rely on strong academic literature to inform policy decisions around the future of work.

## Introduction

As governments around the world move away from in-house policy expertise, they have grown increasingly reliant on the advice of global management consulting firms (GMCFs) and International Organizations (IOs) [1]. These organizations inform the policy process by generating a mass of policy reports that fall under the label of grey literature, which is literature that is “not subject to traditional academic peer‐review processes” [2, p. 433]. Given the nature of this type of literature, it does not appear in scholarly databases which makes conducting citation analysis difficult. Citation analysis is a bibliometric technique that uses citation linkages between publications on a particular topic to investigate how an evidence base has formed over time and evaluate the quality of this evidence base. However, considering the growing political clout that the organizations that publish this kind of literature have, there is a pressing need to use such techniques to evaluate the work that informs their recommendations.

In this paper, we use citation analysis to focus on patterns of the referencing in publications by GMCFs and IOs on the timely topic of “future skills” or “21^st^ century skills” which refer to the new skills needed by workers given the “changing nature of work in an era marked by disruptive technologies” [3]. Interest in this topic has intensified since the beginning of the fourth industrial revolution [4], which started around in the mid-2000’s enabled by extraordinary technology advances. Over the last two decades, this topic has generated reams of policy reports published by some of the largest IOs (e.g. OECD, ILO and World Bank) and GMCFs (e.g. Accenture, Deloitte and McKinsey) working in the area, which all fall under the purview of grey literature.

In another publication, we examine the epistemic authority of IOs and GMCFs their generation of what Peck and Theodore [5] label “fast policy,” a process in which decision-making occurs at a high velocity with the potential for generating poorer quality information in this policy area [5]. In this paper, we evaluate the referencing patterns in grey literature documents published by GMCFs and IOs. We examine reference counts, rates of reliance on scholarly literature and rates of self-referencing by institutions. Furthermore, we compare referencing practices both within and across GMCFs and IOs to examine whether research may function differently in these organizations. Lastly, we identify the key scholarly and grey publications that inform the evidence base behind this important topic which has been highly influential in government policy creation surrounding education and training programs and policy area in which these types of organizations exert considerable authority and influence [6].

In the following sections we discuss the ways in which grey literature is used in policy development, introduce the topic of future skills, and introduce metrics (institutional self-referencing, type of literature and reference counts) commonly used to analyze references in citation analysis.

## Grey literature and policy development

Government policies around the world have been increasingly influenced by GMCFs and IOs for often different reasons. As local governments in advanced economies have identified their own skill and knowledge limitations around policy development, they have become increasingly reliant on the research and policy expertise of consultants [1,7]. International organizations, too have gained influence on public policy either by controlling funding, developing diplomatic relationships or through their perceived legitimacy on matters of economic and policy expertise [3].

The research produced by these organizations largely falls under the umbrella of grey literature [8,9]. Grey literature is defined as “that which is produced on all levels of government, academics, business and industry in print and electronic formats, but […] not controlled by commercial publishers, i.e. where publishing is not the primary activity of the producing body” [10, p. 10] or “the diverse and heterogeneous body of material that is made public outside, and not subject to, traditional academic peer‐review processes” [2, p. 433]. While, historically, access to this grey literature has been elusive, more recently, digitization has increased their availability and, therefore, their influence in both scholarly publications and public policy [9].

Considering the influence of GMCFs and IOs on domestic and international policy, it is important to recognize how their research differs from academic/scholarly research. Research by non-academic organizations and scholarly research are vastly different in terms of objectives, theory utilization and data collection. While scholarly researchers are motivated by a search for the “truth” policy or non-academic research focuses on practical real-world applications [11]. Research by GMCFs requires the production of specific deliverables for clients, while research by IOs focuses on solving public problems and informing policy [11,12]. Scholarly research, in contrast, places an emphasis on transparency, replicability and novel contributions to the academic literature [12]. Academic researchers must also operate under the expectation that all aspects of their research can be and often are scrutinized under a blind or double-blind peer-review process and, therefore, all their suggested implications must be defensible. Non-academic research, on the other hand is not required to be reported in a manner that allows for replication, is often generalized beyond the research context and if peer-reviewed, is generally not subject to processes such as blind reviews that are designed to promote impartiality [8,12].

## What are future skills?

The Fourth Industrial Revolution, a term coined by Klaus Schwab, describes a paradigm shift brought about by technologies that is already in place and is forecast to be unprecedented in “velocity,” “breadth and depth” and global impact [4, p. 8]. Schwab [4] identified three types driving forces in the fourth industrial revolution: physical, digital, and biological. The common factor between all the drivers, however, is that they all “leverage the pervasive power of digitization and information technology” [p. 19].

The proliferation of these technologies has brought with it seismic shifts in a number of areas including the labour market. Whether this disruption will result in a dichotomy in which humans and machines will compete for work, however, is often debated in media and academic circles [13]. It is not clear whether jobs in whole or only aspects of jobs and specific tasks will be replaced by machines. Even in the worst-case scenario, it is likely that although there will be a displacement effect whereby machines will replace human workers in traditional occupations, as with previous industrial revolutions, there will likely be new types of occupations. The most optimistic forecasts are that human and computerized contributions will be complementary [4,13,14].

The non-technologically driven COVID-19 pandemic has also led to massive disruptions to the labour market by accelerating the shift toward remote and other technology-mediated forms of work [15]. By May 2020, approximately half of employed workers in the United States had moved to working online [16]. A major question that arises with this shift in the global socio-economic architecture is how to support or enable individuals to acquire the skills needed to succeed in the evolving market. In this context, the concept of “future skills” has changed to “now skills” needed for employment success.

Emerging literature has now begun to identify the skills required for success in this developing reality. While technologies can substitute routine tasks with relative ease, tasks that require innovation, problem-solving, social intuition and complex communication have been particularly challenging to automate [14,16,17].

These tasks involve the use of complex or “expert thinking” which is defined as thinking that utilizes innovation when traditional solutions are no longer appropriate. Such processes cannot be translated into algorithms or heuristics and require the ability to improvise, a skill that automation is yet to master [14].

### Who is informing policy around future skills?

Across disciplines, the skills needed for the future world of work have been identified using a variety of terminologies, such as future skills, 21^st^ century skills, digital aptitudes, survival skills [18] and skills needed for the Fourth Industrial Revolution or Industry 4.0 [4]. Several organizations have also been created with the sole mandate to teach these skills; however, there is little consensus on the definitions of the skills needed for success in an increasingly automated world. In fact, in a survey of 17 countries, most countries stated that they either did not have national definitions for such skills or that these skills were not clearly defined [19].

In addition to future skills-specific organizations and academics, the field has been shaped to a great extent by two types of influential policy actors: international organizations (IOs) and global management consulting firms (GMCFs), both of whom have published extensively on this topic over the past few decades. It is widely accepted in the international relations and public policy literature that IOs and GMCFs influence governments in a variety of ways [20–24]. For an in-depth discussion of the authority held by IOs and GMCFs in the future skills sector specifically, see White et al [3].

Even though findings from reports published by both GMCFs and IOs organizations have informed policy and have started shaping the pedagogy at numerous educational institutions [6,22]; there is limited understanding about how and from where their claims emerge.

It is well-established that reflecting and building upon existing literature is an integral part of the research process and authors reference prior publications to link their work to relevant research in the field [25,26]. However, very little is known about the literature that IOs and GMCFs reference when they make recommendations about future skills. In light of the impact that these publications may have on education and policy going forward, it is imperative then that we examine the references relied on by these two influential actors. Furthermore, an investigation of differences, if any, in the patterns of referencing across these two types of organizations can help inform audiences that rely on these reports for decision-making.

## Examining referencing patterns using citation analysis

Citation analysis is a method used to investigate linkage patterns between publications to identify influential works in a field of study [26,27]. Citation analysis can be used to evaluate the citation practices in a body of research evidence by examining the number of sources cited by a document, the types of sources cited and rates of self-referencing [28]. The term citation analysis covers an examination of both citations (sources in text) and references (sources in bibliographies). However, since we specifically examined the bibliographies of these reports, we use the term “references” in this paper.

In this paper we use citation analysis to investigate 1) referencing practices in reports published by GMCFs and IOs and 2) trends in referencing patterns across these publications.

### Number of sources cited

The simplest aspect of referencing practices that can be examined using citation analysis is the number of sources referred to in a document. The use of references to back up key assertions demonstrates that authors are well-situated in the literature, provides credibility to their viewpoints, and can lend support to their claims and findings [29,30]. Indeed, as students are taught formal academic writing, they are often provided with guidelines around the minimum number of sources they must rely on to teach them informational literacy, and how to establish credibility [31,32].

The number of references in a paper has also been correlated with the projected influence of the paper itself, with findings across disciplines showing that papers that cite more references also tend to get cited more themselves [33,34]. However, the number of sources cited in a publication is determined by various factors, including the discipline, amount of research in the field, depth of the literature review, and journal requirements [35]. For example, Halevi [36] found that, on average, the social sciences, physics and astronomy had approximately 54 references per article. Health professions and Earth and Planetary Sciences on the other hand, cited approximately 8 and 17 references per article, respectively [36]. To our knowledge, there are no specific referencing thresholds in non-academic writing. However, given the importance of citing sources and building upon existing literature, it may be argued that having more references is reflective of better referencing practices.

### Types of sources

Citation analysis also allows us to investigate the impact of different types of sources, and whether a citation network is based primarily on scholarly or grey literature. Scholarly sources that are academic and peer-reviewed undergo a vetting process and are held at a higher standard than grey sources, which do not undergo any critical appraisal [37]. The lack of formal standards of reporting in grey literature further imposes limits on the generalizability and replicability of their findings [38]. For example, authors of meta-analyses exclude grey literature to maintain the quality of the work that is included [39]. While it cannot be argued that scholarly work does not have its own drawbacks (for example, publication bias), the lack of oversight and rigorous review of grey literature has cast some serious doubts about its legitimacy [40].

### Self-citation

Citation analysis can also be used to investigate bias in the literature in a field of study. For example, various citation analyses of scholarly literature have found that there is an excessive amount of self-citation in some scientific articles, with authors or organizations citing more of their own previous publications than any other sources [41,42]. Jahani & Yaminfirooz [43, p. 401] define synchronous self-citation as “… the percentage ratio of citations given by an author to his/her previous works to all citations in the paper at hand. For example, if an author publishes a paper with 5 citations of which 2 papers belonged to him/her, the ratio of his/her synchronous self-citation would be 2/5 or 40%”.

In peer-reviewed journal articles, synchronous self-citation can range from 7% to 31% of an article’s references, with over 20% being considered excessive [42,44]. To our knowledge, no such threshold has been established for grey literature. However, the 20% threshold was used to evaluate self-citation at the institutional level in the university context [45]. Given this lack of guidance specifically for grey literature we use the 20% standard from past work. Excessive self-citation can have serious implications for the influence an article, author, journal or institution may have in a field [44]. A high amount of self-citation can indicate a biased viewpoint, with authors and institutions building over emphasizing their own prior work and not addressing contradictory findings [46].

To date, only a handful of studies have examined the impact of self-citation at the institutional level, and have primarily focused on universities [47–49]. For example, Hendrix [47] suggests that high rates of self-citation at universities may be indicative of “citation circles” where researchers or research groups purposely try to inflate their bibliometric indicators. In a study of self-citation in Iranian universities, Jahani and Yaminfirooz [43] found that non-medical universities in Iran had significantly higher rates of self-citation than medical universities. Yan and Sugimoto [48] examined institutional self-citation in library and information sciences (LIS) in the United States and found that self-citation rates differed by the specific LIS-related topics on which universities focused. Although in-depth research at the institutional level is limited, high rates of self-citation in publications produced specifically by institutions such as GMCFs and IOs may be of concern when considering: 1) the political clout held by certain institutions and organizations; and 2) the importance of incorporating a broad range of perspectives when developing wide-ranging policies.

### Using citation analysis to investigate patterns

In addition to assessing referencing practices in a field of study, citation analysis can also be used to identify the publications that have had the most influence in shaping the knowledge in that field. Citation patterns can be presented descriptively as visual networks, with publications represented as nodes and referencing patterns represented as the connections between them. Citation analysis can also be used to assess the contributions of publications over time. Prior research has shown that most references of academic works occur three years after publication [49]. Grey literature, on the other hand, is referenced soon after publication and becomes obsolete over a shorter period of time [50]. The implication of this compressed knowledge diffusion period may result in what is known as “fast policy,” a topic explored in further detail in White et al [3].

### Challenges with citation analyses of grey literature

Citation analysis is most commonly used to create citation networks between scholarly peer-reviewed publications and is facilitated by databases such as Scopus and Web of Science. Less common, however, are citation analyses using grey literature, which have been primarily conducted in the fields of agriculture and physics [51,52]. One reason for this that authors of citation analyses in grey literature note is that, in contrast to scholarly literature, the lack of established referencing styles in the grey literature creates a substantial challenge for using this analysis [51,52]. Unsurprisingly, in terms of practices, findings from prior citation analyses suggest that grey literature publications tended to cite *more* grey, and scholarly publications tended to cite *less* grey literature [50].

## Current study

In this study we use citation analysis to examine the growth over time and interconnectivity between grey publications about the skills needed in the future world of work that IOs and GMCFs produce. In particular, we investigate: 1) the extent to which these organizations use references; 2) whether they utilize data from other sources or rely primarily on findings from their own research; and 3) whether they rely heavily on scholarly sources or other grey literature. Furthermore, given that GMCFs produce research as deliverables to specific clients and stakeholders, and IOs aim to inform public policy, we investigate differences in referencing patterns across these two types of organizations. To our knowledge, this is a novel contribution to the field and there have been no prior citation analyses that compare the types of publishing organizations.

Our research questions are:

1. *How extensive are the references used in non-academic publications on skills needed in the future world of work*? *Is there a difference in the number of sources referenced by GMCFs and IOs*?
2. *Do non-academic organizations that publish literature on skills needed in the future world of work engage in high levels of self-referencing*? *Is there a difference in self-referencing rates across GMCFs and IOs*?
3. *To what extent does scholarly literature contribute to the referenced works in comparison with grey literature*? *Is there a difference in scholarly referencing rates across GMCFs and IOs*?
4. *What are the most referenced grey and scholarly sources in the non-academic literature on skills needed in the future world of work*?

We hypothesize that in keeping with prior findings of citation analyses in grey literature, non-academic organizations will rely more heavily on grey sources in comparison to scholarly sources. However, given that their findings are public, have widespread policy implications and are more open to scrutiny, we hypothesize that IOs will rely on more scholarly and overall sources than GMCFs. We hypothesize that most organizations will engage in high levels of self-referencing, but these rates will be lower in IOs. Lastly, we hypothesize that the influential scholarly literature cited by these organizations will be cited for longer than the grey literature.

## Methods

### Identification of sources

#### Global management consulting firms (GMCFs)

Although numerous consulting firms may be publishing on future skills, our search strategy focused on credible GMCFs that have been influential in global policy. Research suggests that a firms’ reputation and ranking are important determinants of clients’ decisions to hire them [53] and comply with their reccomendations [54]. Therefore, we used rankings from the reputable [55,56] market research agency, Vault, to select the top ten “most prestigious” global management consulting firms in 2020 [57]. In descending order, these firms were McKinsey, BCG, Bain, Deloitte, PwC, Booz Allen Hamilton, EY, Accenture, KPMG, and Oliver Wyman. Numerous studies have examined the impact that these GMCFs have had on policy and reforms [22,58–61]. Three consulting firms (Bain, Booz Allen Hamilton and Oliver Wyman) were removed from this list because an initial screening found fewer than five relevant publications.

#### International organizations (IOs)

International Organizations were identified by focusing on the UN and agencies under its purview with publications on our key terms. Organizations identified through this process were the International Labour Organization (ILO), United Nations Economic and Social Council (ECOSOC), the United Nations Educational, Scientific and Cultural Organisation (UNESCO), and the World Bank. The search was expanded to other major IOs that published on employment and labour markets in major industrialized nations. Through this process the Organisation for Economic Co-operation and Development (OECD) and the European Union (EU) were included. The World Economic Forum was also included in this study even though it is a non-governmental organization because of its extensive publications on the topic.

### Collection of reports

Websites of all selected firms and organizations were searched for using key search terms and synonyms related to “future skills” and “future of work”. The most recent publications were collected from the organizations’ websites. The digital archive tool, the Wayback Machine by the Internet Archive, was used to collect archival data from their websites back until the year 2000. This process was supplemented to include other relevant documents by using a regular search engine to search the name of the organization and expressions referring to the skills needed in the future world of work, such as “future skills,” “21^st^ century skills,” and the “future of work.” Through this process 318 documents were identified. Each document was read in its entirety and included if it referred to skills or aptitudes that workers should acquire for success in the 21^st^ century labour market. We additionally removed reports that contained no references or those that only discussed skills needed in the future world of work in passing, resulting in a database of 234 policy reports (Supporting Information). In this database, a unique identifier was assigned to each report, and the following data was extracted: author(s), the publishing organization, year of publication.

### Extraction of references from policy reports

To account for differences in formatting and allow for comparability, word counts and standardized page counts were computed for each document. References were then manually extracted from the bibliography of the document, resulting in a database with 15,479 references. Three hundred and thirty-five references (0.02% of the database) belonged to documents that were not published in English and were removed from the database, resulting in a database with 15,144 extracted references. Each extracted reference was labelled with: the identifier of the policy report from which it was extracted, authors (individuals or organizations), year of publication, publishing source, type of reference (scholarly, grey or other) and type of document. References were classified into three types: scholarly, grey, and other.

Scholarly sources were defined as peer reviewed, predominantly produced by academics and published by a reputable academic publisher, with academics as the intended audience. Types of documents in this category included journal articles, dissertations, and academic books. Grey literature was defined as content that was not controlled by commercial publishers, and could be published by policy institutes, consulting firms, businesses and public or private corporations. Examples of types of documents in this category include reports, working papers, conference papers and government documents. As working papers can include pre-publication versions of scholarly documents, when possible, we located the final scholarly versions of these documents; otherwise, they retained the working paper classification under grey literature [62,63].

The ‘other’ category included other material such as links to websites, blogs and material produced by commercial presses or magazines that was intended for non-scholarly audiences. Types of sources in this category included textbooks, magazine articles, webpages, blogs, and newspaper articles.

In our analyses, we examined only references that were scholarly or grey literature and therefore 3117 references categorized as “other” were removed from the database, leaving us with a sample of 12,176 scholarly and grey references, 90% of which were published after the year 2000. References were then examined for duplicates using fuzzy matching logic using the matchit command in Stata v.15. This command provides a similarity score between two different text strings by performing a number of different matching techniques. Once the similarity score was created by the Stata command, “duplicates” that had a similarity score of greater than 50 percent were visually compared by the researchers and were manually determined to be a duplicate or not. Each unique reference was then assigned its own identifier.

### Data analysis

All statistical analyses were conducted using SPSS v.27.

1. *How extensive are the references used in non-academic publications on skills needed in future world of work*? *Is there a difference in the number of sources referenced by GMCFs and IOs*? Descriptive data are presented for reference rates across all organizations. Rates of referencing were compared across GMCFs and IOs using a Mann-Whitney U Test (appropriate for non-normally distributed data). For comparability, the rate of references per word was computed for each report and similarly compared across GMCFs and IOs.
2. *Do non-academic organizations that publish literature on skills needed in the future world of work engage in high levels of self-referencing*? *Is there a difference is self-referencing rates across GMCFs and IOs*? Instances in which an organization cited work that they themselves had published were coded as self-references. The rate of self-referencing was computed for each document as the proportion of total references that were published by the same organization. A Mann-Whitney U Test was conducted to determine whether self-referencing rates differed across GMCFs and IOs.
3. *To what extent does scholarly literature contribute to the referenced works in comparison with grey literature*? *Is there a difference in scholarly referencing rates across GMCFs and IOs*?

Citation analysis literature recommends setting a threshold of number of references for inclusion in analyses used to determine which sources are influential in a field of study [64,65]. We utilized a conservative threshold of at least 2 references and removed all citations that only occurred once in the database. This resulted in a database with 810 unique references. For each report, the scholarly-reference rate was computed as the proportion of total references in the report that were identified as scholarly. A Mann-Whitney U Test was conducted to determine whether scholarly-reference rates differed across GMCFs and IOs.

## Results

### Number of references

Table 1 presents descriptive information about the occurrence of references in the bibliographies of the selected organizations. A series of Mann-Whitney U tests was conducted to determine whether there were differences between GMCFs and IOs on various indicators. Results of these tests are shown in Table 2. The median number of references was statistically significantly higher for IOs than for GMCFs. As a follow-up using a standardized approach, differences in number of references per word were tested across both types of organizations. Findings similarly showed that the number of references per word was statistically significantly higher in IOs compared to GMCFs. Furthermore, the length of documents (measured by total number of words) published by IOs was also statistically significantly higher than GMCFs.

**Table 1:** Descriptive data on reference-rates across organizations.

Organization	Type of Organization	Number of Reports	Mean Refs	Mean Word Count	Mean Refs/Word^a^	SD Refs/Word	Range Refs/Word
Accenture	GMCF	20	9.25	7724.10	1.37	0.84	0.30 to 3.44
BCG	GMCF	3	18.33	14771.67	1.19	0.67	0.42 to 1.67
Deloitte	GMCF	27	26.07	17846.48	1.64	1.30	0.02 to 6.87
EY	GMCF	1^b^	4.00	7934.00	0.50	NA	NA
KPMG	GMCF	5	5.00	13368.80	0.46	0.43	0.10 to 1.17
McKinsey	GMCF	16	58.19	34176.81	1.95	0.79	0.77 to 3.70
PwC	GMCF	7	20.29	9747.14	1.64	1.24	0.59 to 4.15
European Union	IO	22	48.50	30674.45	1.90	1.58	0.43 to 6.43
ILO	IO	57	60.02	27909.12	4.00	2.93	0.10 to 12.69
OECD	IO	40	76.00	26978.08	4.72	6.38	0.49 to 40.82
UN	IO	1^b^	33.00	14324.00	2.30	NA	NA
UNESCO	IO	15	41.53	38152.73	2.75	2.81	0.00 to 11.11
WEF	IO	15	41.93	28874.20	1.96	1.15	0.11 to 4.78
World Bank	IO	5	264.20	57934.20	5.33	1.84	3.02 to 7.31

^a^For interpretability, references per word have been multiplied by a factor of 1000.

^b^These organizations only had one report with references, therefore their findings should be interpreted with caution.

**Table 2:** Mann-Whitney U test of the number and references, number of references per word and total number of words across IOs and GMCFs.

	Type of Organization	No. of Reports	Median	IQR	Mean Rank	U	Z-value	p-value
**Number of references**	IO	155	41.00	56.00	136.48	3180	-6.01	0.000
	GMCF	79	13.00	28.00	80.25			
**Number of references per word**	IO	155	0.00280	0.00340	135.67	3306	-5.75	0.000
	GMCF	79	0.00130	0.00130	81.85			
**Total number of words**	IO	155	17058	28755	126.03	4800	-2.78	0.007
	GMCF	79	10406	13166	100.77			

### Institutional self-referencing

**Fig 1:**
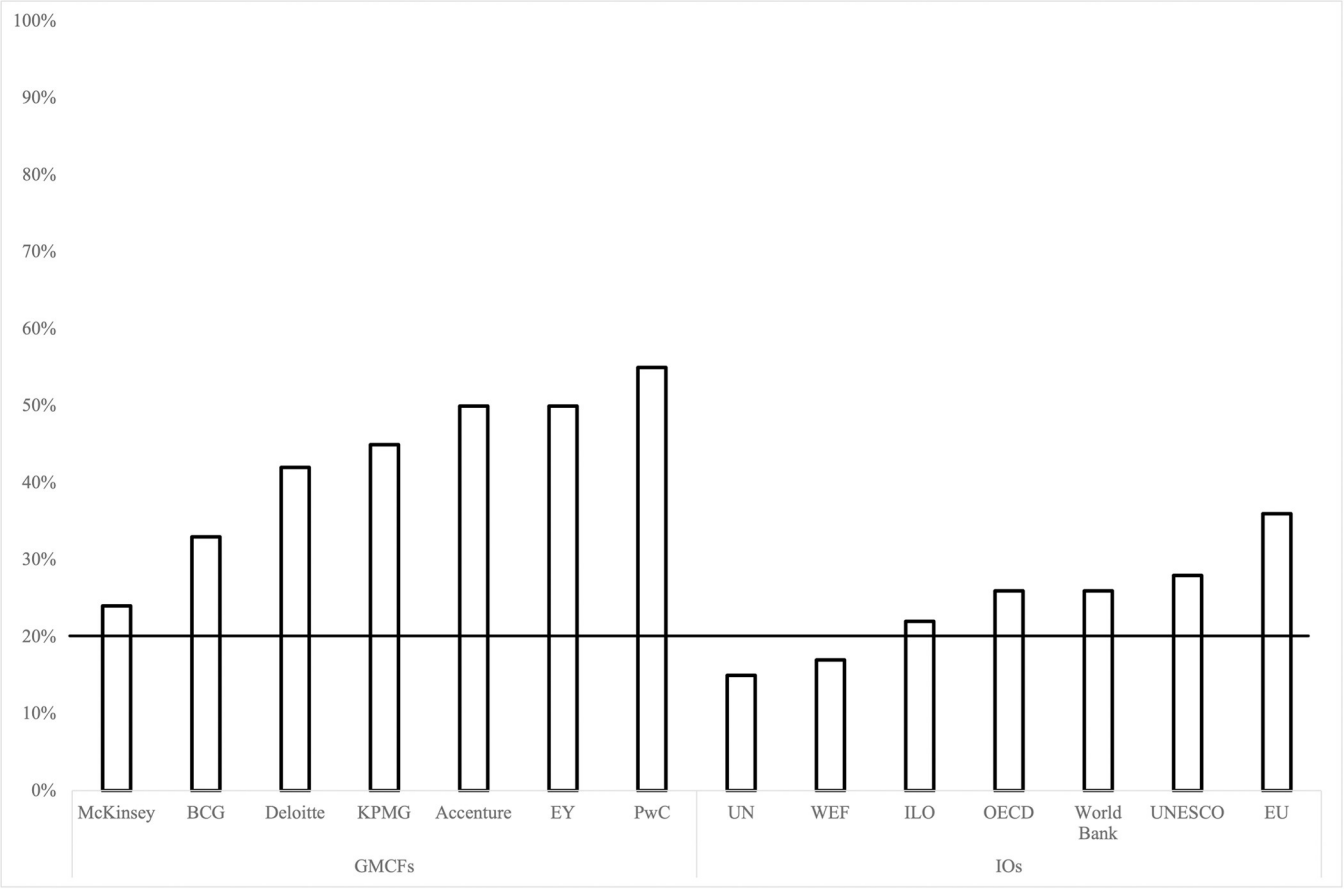
Rates of self-referencing across organizations. Note Fig 1. The horizontal line indicates the 20% threshold for excessive self-referencing.

Note that although the literature refers to this phenomenon as self-citation, we use the term self-referencing to remain consistent and indicate that we are referring to bibliographic references and not in-text citations. As shown in Fig 1, that depicts rates of self-referencing (Y-axis) for each GMCF and IO (on the X-axis), all the GMCFs and the majority of the IOs engaged in self-referencing at a rate higher than the threshold of 20%. While there is a degree of overlap between organizations, for example between UN agencies such as the ILO and UNESCO, we adopted a conservative approach in our analyses by considering them to be independent organizations, therefore their rates of self-referencing may be higher than presented here.

A Mann-Whitney U test was conducted to determine whether there were differences in self-referencing rates across the two types of organizations. Findings of this test (shown in Table 3) suggest that self-referencing rates of GMCFs were statistically significantly higher than those of IOs.

**Table 3:** Mann-Whitney U test of rate of self-referencing across IOs and GMCFs.

	Type of Organization	No. of Reports	Median	IQR	Mean Rank	U	Z-value	p-value
**Rate of self-referencing**	IO	155	0.18	0.30	146.62	8423	4.70	0.000
	GMCF	79	0.38	0.36	102.66			

### Citation network of scholarly and grey sources

**Fig 2:**
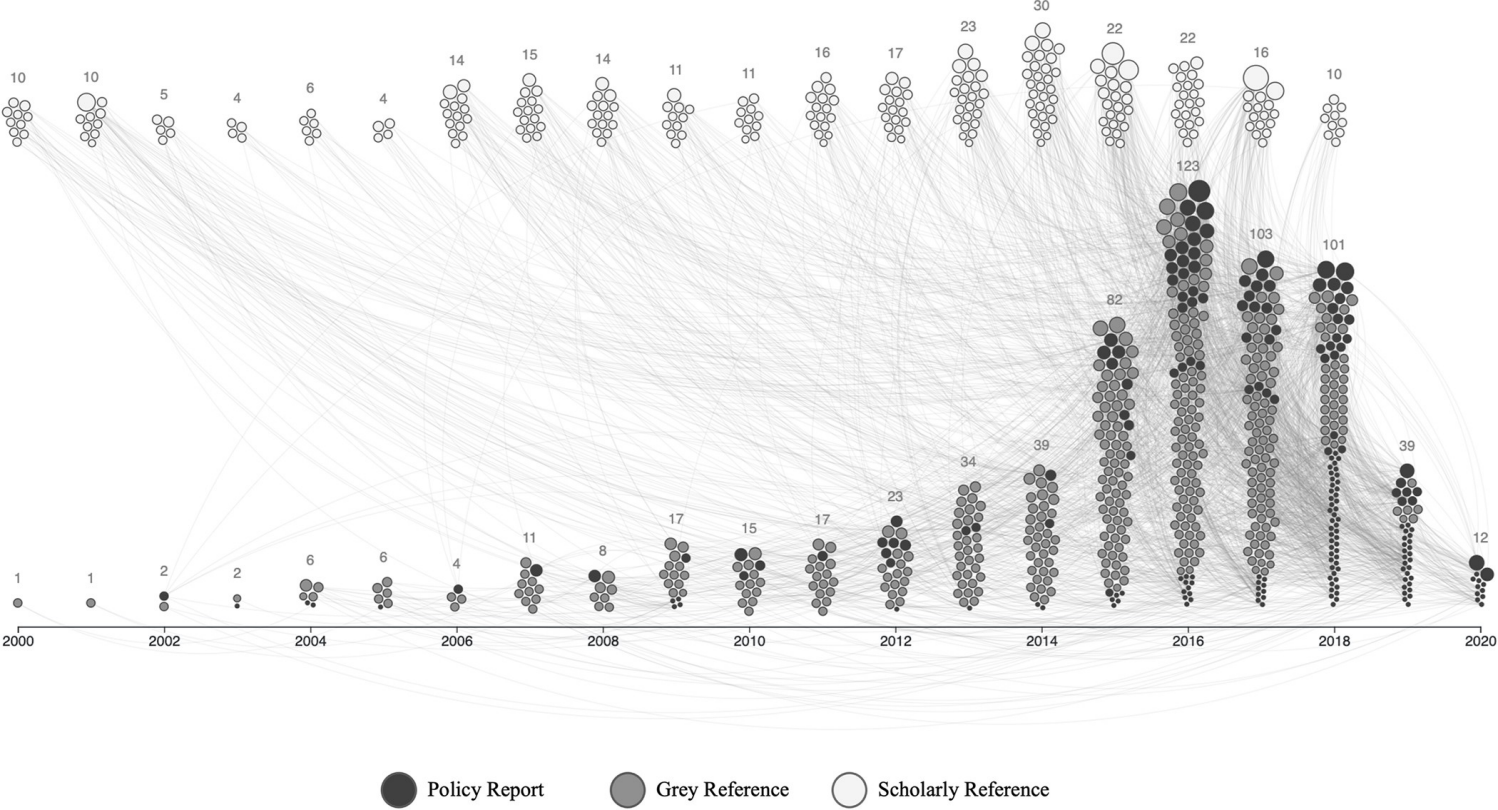
Citation network timeline of scholarly and grey sources referenced at least twice in our database.

We used the JavaScript library D3 to create a network timeline (Fig 2) at two-year intervals that shows the linkages between the policy reports and references that occurred at least twice. This network is separated by the type of literature with the top half of the graphic depicting scholarly publication (white nodes) and the bottom half depicting grey literature publications. Within grey literature, we present our policy reports (dark grey nodes) and other grey literature (light grey nodes) that the primary reports refer to. The size of the nodes in this graphic is based on the number of times each publication is referenced, with larger nodes (publications with more references) clustering at the top.

In the early years of publication in this field, from 2000 to 2008, these organizations referenced more scholarly than grey publications, after which for about three years they were equivalent. After 2012, when the number of publications on the future of work began to rise, more grey than scholarly publications were referenced. This gap widened substantially in 2015, after which the number of grey publications increased rapidly and reached more than double that of scholarly publications. As can be seen by the density of connections, in the last decade the contribution of grey literature referenced by these organizations supersedes that of scholarly literature. This gap that has become substantially more pronounced over the last five years.

It is important to note that the tapering of the timeline in 2020 is not due to a reduction in publications, but rather it reflects the period captured in our data collection which took place from July 2019 to February 2020.

**Fig 3:**
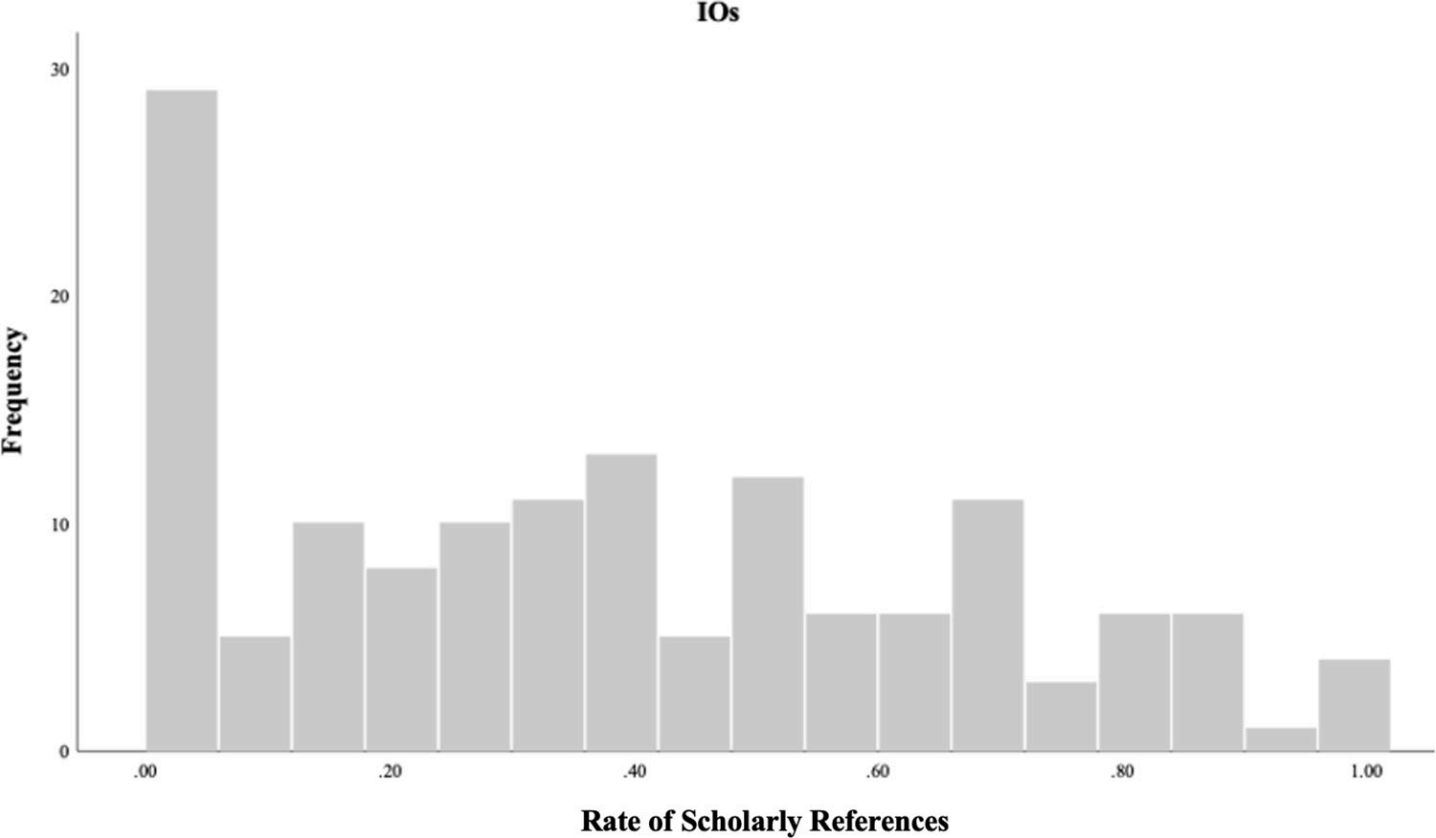
Distribution of scholarly references by IOs.

**Fig 4:**
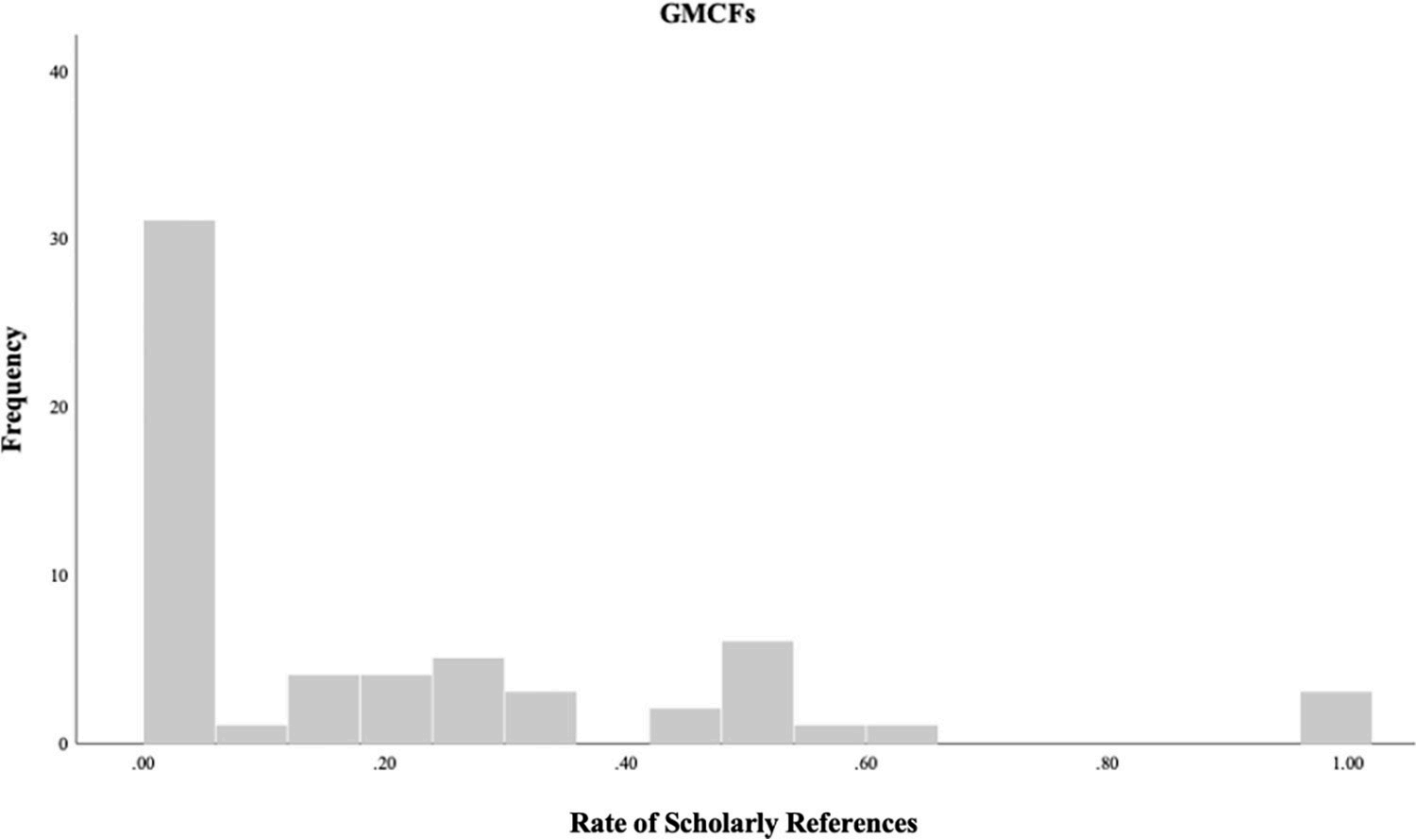
Distribution of scholarly references by GMCFs.

Figs 3 and 4 depict the distributions of rate of scholarly references (ranging from 0 to 1 on the X-axis) by the frequency counts of reports (on the Y-axis) by IOs and GMCFs, respectively. For both IOs and GMCFs the highest frequency occurs when rate of scholarly references is zero. As shown in Fig 3, the frequency of reports by IOs varies across the rates of scholarly references, whereas as Fig 4 with GMCFs shows there is less variability in rate of scholarly references. A Mann-Whitney U test (Table 4) showed that rate of scholarly references were statistically significantly higher for IOs than GMCFs.

**Table 4:** Mann-Whitney U test of rate of scholarly references across IOs and GMCFs.

	Type of Organization	No. of Reports	Median	IQR	Mean Rank	U	Z-value	p-value
**Rate of scholarly references**	IO	146	0.36	0.46	116.09	2689	-4.55	0.000
	GMCF	51	0.00	0.31	75.08			

### Profiles of most frequently cited sources

Tables 5 and 6 present the profiles of the most influential scholarly and grey sources referenced by these non-academic organizations. The majority of these sources were published after 2010 and most of the influential scholarly sources were published in economics journals. Four of the most influential grey publications belonged to our pool of policy reports.

**Table 5:** Most frequently referenced scholarly sources.

Title	#	Author	Year	Type	Source
The Future of Employment: How Susceptible Are Jobs to Computerisation?	45	Frey, C. B., & Osborne, M. A.	2017	Journal Article	Technological Forecasting and Social Change
The Skill Content of Recent Technological Change: An Empirical Exploration	32	Autor, D., Levy, F., & Murnane, R. J.	2003	Journal Article	The Quarterly Journal of Economics
Why Are There Still So Many Jobs? The History and Future of Workplace Automation	25	Autor, D.	2015	Journal Article	Journal of Economic Perspectives
The Second Machine Age: Work, Progress, And Prosperity in A Time of Brilliant Technologies	19	Brynjolfsson, E., & Mcafee, A.	2014	Book	Norton
Skills, Tasks and Technologies: Implications for Employment and Earnings	19	Acemoglu, D., & Autor, D.	2011	Journal Article	Handbook of Labour Economics
The Growing Importance of Social Skills in The Labor Market	18	Deming, D. J.	2017	Journal Article	The Quarterly Journal of Economics
Putting Tasks to the Test: Human Capital, Job Tasks, and Wages	12	Autor, D. H., & Handel, M. J.	2013	Journal Article	Journal of Labor Economics
The Rise and Nature of Alternative Work Arrangements in the United States, 1995–2015	12	Katz, L.F. & Krueger, A. B.	2018	Journal Article	Industrial and Labor Relations Review
Explaining Job Polarization: Routine-Biased Technological Change and Offshoring	11	Goos, M., Manning, A., & Salomons, A.	2014	Journal Article	American Economic Review

**Note:** Sources referenced more than 10 times in the dataset are presented.

**Table 6:** Most frequently referenced grey sources.

Title	#	Author	Year	Type	Source
The Risk of Automation for Jobs in OECD Countries: A Comparative Analysis*	30	Arntz, M., Gregory, T., & Zierahn, U.	2016	Working Paper	OECD
Non-standard Employment Around the World: Understanding Challenges, Shaping Prospects	18	ILO	2016	Report	ILO
Automation, Skills Use and Training*	17	Nedelkoska, L., & Quintini, G.	2018	Working Paper	OECD
The Future of Jobs: Employment, Skills and Workforce Strategy for the Fourth Industrial Revolution*	16	WEF	2018	Report	WEF
A Future That Works: Automation, Employment, and Productivity*	15	Manyika, J. et al.	2017	Report	McKinsey
World Development Report 2016: Digital Dividends	14	World Bank	2016	Report	World Bank
A Labor Market That Works: Connecting Talent with Opportunity in the Digital Age	14	Manyika, J. et al.	2015	Report	McKinsey
Jobs Lost, Jobs Gained: Workforce Transitions in a Time of Automation	12	Manyika, J. et al.	2017	Report	McKinsey
Labour Market Mismatch and Labour Productivity: Evidence from PIAAC Data	12	McGowan, M. A., & Andrews, D.	2015	Working Paper	OECD
The Future of Jobs Report 2018	12	WEF	2018	Report	WEF

**Note:** Sources referenced more than 10 times in the dataset are presented. Sources marked with an asterisk (*) belong to our pool of policy reports.

## Discussion

Noting the influx of non-government and non-academic actors in the policy sphere, we investigated the bibliographic component of research published by these organizations. We used citation analysis, a tool used primarily to evaluate academic literature, to identify the influential sources in the field, and evaluate referencing practices of the grey literature published by influential organizations on the skills needed for the future world of work. Using Vault rankings and agencies associated with the UN, we identified the most prestigious GMCFs and IOs and generated a collection of 234 reports on this topic.

The mean number of reports per organization was 14, however organizations contributed as little as 1 to as many as 57 reports. Reports were included in our study if they were relevant to the key topic and contained references. Therefore, some of our purposely selected organizations such as EY, UN and BCG ended up having three or less reports. Findings from these organizations should be interpreted with caution because they contributed few data points to the analyses.

The mean number of references per organization ranged from as low as four to as high as 264. Given the differences in publication lengths, comparisons should be made accordingly and therefore standardize these values by page or by word. In academia, the number of appropriate citations varies by disciplines, journals and the range of literature on the topic being investigated [37]. To our knowledge, there are no specific referencing thresholds in non-academic writing, and we indeed found that references lengths vary considerably across publications within and across organizations.

We found that the majority of the non-academic organizations that publish on the future of work engage in levels of self-referencing that are considered excessive by academic standards. These rates reached as high as 55% and 50% for PwC and Accenture respectively. The high (50%) self-referencing rate for EY may be explained by the fact that they had only one report that included references and a very low reference-rate in general, they only cited four sources, two of which were published by their organization. Although overall IOs had lower self-referencing rates than GMCFs, they too were above the recommended threshold for scholarly work, with rates as high as 36% and 28% for the EU and UNESCO respectively.

Rates of self-referencing vary within academic literature, but bibliographic research generally acknowledges the dangers this practice poses in terms of inflating the influence of a particular publication or the impact factor of a journal. This practice is kept in check in academic writing, with researchers being banned from journals [66] and journals being banned from ranking lists [67] as a result of findings of self-citation abuse. The lack of such regulations in non-academic research means that publications that heavily self-cite may be regarded as more influential than they really are. Given the influence these organizations exert on policy, our findings flag an important concern.

In keeping with findings from citation analyses of grey literature, we found that most of these non-academic organizations relied more heavily on grey than scholarly literature. Considering that lack of empirical rigour and transparency in grey literature, findings from these documents must be interpreted with caution and may have limited generalizability. Exceptions to this trend, however, were the World Bank and OECD, both of which relied primarily on scholarly findings. It is not surprising that these IOs had a significantly higher reliance on scholarly literature than GMCFs. Reasons for these differences will be discussed in further detail shortly.

The most influential scholarly sources in our secondary dataset primarily were published over the last decade and primarily came from economics journals. These findings highlight the need for a more diverse and interdisciplinary approach to the understanding and development of future skills, and the inclusion of sociological, psychological and anthropological perspectives among others. All of the most influential grey publications were documents published within the list of organizations we had identified, lending further credence to our search strategy. The majority of the influential grey publications belonged to the IOs with widespread international reach. White et al [3] explores how the life cycle of these publications may reflect “fast policy” and the epistemic authority of male economists in more detail.

Overall, we found that by comparing both in absolute terms, and after standardizing, IOs produced longer documents, referenced more literature, engaged in less self-referencing and utilized more scholarly literature than GMCFs. Since, to our knowledge, there have not been any prior comparisons of referencing patterns in publications by these organizations, we can only speculate as to why this may be. The IOs that we identified work with national governments and influence policy decisions, and therefore may experience greater scrutiny than their private counterparts. They are also more likely than GMCFs to employ academics to conduct research on their behalf [68,69]. Furthermore, the World Bank, OECD and ILO publish working papers that are often pre-publication versions of future journal articles and may therefore be motivated to cater to academic audiences. These organizations also collect large-scale data (for example PIAAC by OECD) that are often used by academics, creating a larger pool of scholarly literature upon which they can draw. On the other hand, the motivation behind producing these publications may differ for GMCFs which are for-profit organizations and therefore may either publish these reports as part of paid consultancies or may use these reports as marketing tools to attract more clients. A review of the research conducted by consulting firms suggests that it may occur in a feedback loop, whereby methods and tools from ongoing projects create the knowledge base from which future projects are derived [67]. Their practices and methods are therefore constrained to a particular “thought world and its associated language” [70, p. 900].

Connecting our findings back to the discussion of the skills needed in the 21^st^ century workforce, we find that the majority of the influential documents in this domain belong to grey literature and rely on a few key scholarly sources. These documents also engage in high amounts of self-referencing which may over-inflate the credibility of their findings. These findings are problematic in terms of the basis for developing a concrete set of skills around which there is empirical consensus, which in turn poses a roadblock in terms of practical implications. However, considering the need for timely research in this domain it is promising that the majority of these publications are recent, having been published within the last decade. Given the sudden acceleration in automation as a result of COVID-19, it is pressing that empirical data be collected, undergo the peer-review process and ultimately be used to inform skills policy in a post-pandemic world.

### Limitations and future directions

One limitation of our research arises from the lack of publication of report-level citation metrics by GMCFs and IOs. Therefore, we were not able to include indicators of how many times the specific reports were referenced outside of our reference network. However, we focused instead on the influence held by organizations when selecting the policy reports.

Secondly, some of the organizations we investigated are not completely independent of one another, for example the UN, ILO and UNESCO are interdependent. In our analysis, we utilized a conservative approach and considered these organizations to be separate in our exploration of self-referencing. Combining organizations that fall under the same umbrella organization may have resulted in higher rates of self-referencing. Additionally, reports and articles might be referenced by the authors who wrote them as well as the organizations that contracted the authors. As a result, actual rates of self-referencing may be in fact be substantially higher.

In contrast to scholarly literature, conducting such analyses on grey literature presents two major challenges. Firstly, scholarly publications are indexed in databases such as Web of Science and Scopus which export citations in a standardized format and provide article-level metadata (for example, authors’ names and institutions, keywords, country of publication etc.) that can be imported to bibliometric software for quantitative analyses. The lack of such tools for grey literature result in a manual and time-intensive extraction processes and limits the range of analyses that can be conducted. Secondly, despite our substantial efforts to do, we were unable to systematically categorize the reports into distinct categories, an approach that is often used for scholarly work (for example, classifying publications as empirical, methodological or review).

Finally, while a scholarly body of work exists on this topic, we limited our analyses to grey literature published by organizations that are known to have policy influence in this area [3]. We did this because the body of scholarly work that would have to be evaluated in quite large and warrants a separate process/paper. In addition, comparing grey and scholarly work is challenging because grey literature lacks structured reporting standards that are inherent in scholarly work (for example, background and discussion sections that cite comparable literature). Future research using databases that provide article-level metadata should include quantitative analyses of field-specific influence such as reference publication year spectroscopy (RPYS) analysis [71], PageRank analysis and measures of eigenvector and closeness centrality [72]. Investigating referencing practices and influential publications in the scholarly literature will add to our findings and provide a holistic view of literature in this domain.

## Conclusion

We found that, overall, the GMCFs and IOs we examined were inconsistent in the number of sources they referenced, engaged in high levels of self-referencing and relied more heavily on grey literature in comparison to scholarly literature. Upon exploring differences in practices across the types of organizations we found that IOs overall engaged in better referencing practices than global management consulting firms. Considering that the work of these organizations is instrumental in shaping policy around future skills training and pedagogy, our study shows a growing trend of organizations not relying sufficiently on strong academic research and engaging in sub-optimal citation practices to inform their recommendations. These findings call into question the extent to which policy-making in this area is evidence-based.

## Supporting information

S1 File (XLSX)
